# Determining Critical Physiological and Drug Specific Parameters for Enhancing a Physiologically Based Pharmacokinetics Model for the Female Reproductive Tract

**DOI:** 10.3390/pharmaceutics18070903

**Published:** 2026-07-22

**Authors:** An Le, Riyazuddin Mohammed, Junmei Zhang, Lin Wang, Guru R. Valicherla, Phillip W. Graebing, Robert Bies, Lisa C. Rohan

**Affiliations:** 1 Department of Pharmaceutical Sciences, School of Pharmacy, University of Pittsburgh, Pittsburgh, PA 15213, USA; atl55@pitt.edu (A.L.); lwang@mwri.magee.edu (L.W.); gururaghava810@gmail.com (G.R.V.); pgraebing@mwri.magee.edu (P.W.G.); 2 Magee-Womens Research Institute, Pittsburgh, PA 15213, USA; riyazuddinm@upmc.edu (R.M.); junmei.zhang@pitt.edu (J.Z.); 3 Department of Obstetrics, Gynecology, and Reproductive Sciences, School of Medicine, University of Pittsburgh, Pittsburgh, PA 15213, USA; 4 Department of Pharmaceutical Sciences, School of Pharmacy and Pharmaceutical Sciences, University at Buffalo, Buffalo, NY 14214, USA; robertbi@buffalo.edu

**Keywords:** female reproductive tract, solubility, matrix-specific binding, permeability, drug disposition, ex vivo human tissue

## Abstract

**Background/Objectives**: Given the potential for achieving high concentrations at the target site while limiting systemic exposure, delivering drugs directly to the female reproductive tract (FRT) is emerging as a promising strategy for enhancing women’s reproductive health. However, quantitative data describing matrix-specific solubility, matrix-specific binding, and permeability across FRT tissues remain limited, constraining development of physiologically based pharmacokinetic (PBPK) models for intravaginal and intrauterine therapies. **Methods**: Our work was conducted to help fill this critical gap, evaluating four model drugs with diverse physicochemical and transporter profiles, dapivirine (DPV), levonorgestrel (LNG), MK-2048, and 4′-ethynyl-2-fluoro-2′-deoxyadenosine (EFdA; also known as islatravir or MK-8591) in in vitro and ex vivo human models. Plasma solubility, matrix-specific binding in plasma, cervicovaginal fluid, and FRT tissues, and bidirectional permeability across FRT tissues were quantified. **Results**: Our results demonstrated that the plasma solubility varied markedly across compounds, following lipophilicity trends, with DPV (34.14 ± 1.04 µg/mL) and EFdA (1808.02 ± 67.36 µg/mL) exhibiting the lowest and highest solubility, respectively. Hydrophobicity-dependent solubility enhancement by plasma proteins (~2× to >30× higher comparing to aqueous solubility in the literature) was observed for all four model drugs. Apparent binding in plasma, cervicovaginal fluid, and FRT tissues was highly correlated with the model compounds’ lipophilicity, with DPV having the most highly matrix-specific binding (97–99%) and EFdA having the least matrix-specific binding with the greatest variability (15–62%). Regional permeability differed significantly across FRT tissues: the human ectocervix, myometrium, endometrium, and fallopian tubes demonstrated distinct transport patterns consistent with epithelial architecture and the transporter-substrate status of the model compounds. Efflux transporter involvement was evident for MK-2048 and EFdA in Caco-2 models (efflux ratios 2.59 and 7.14, respectively), but was less pronounced in the 3D vaginal model and ex vivo tissues. Across all datasets, permeability and binding were strongly influenced by drug lipophilicity and ionization characteristics. **Conclusions**: Collectively, these findings demonstrate the interplay among solubility, matrix-specific binding, and tissue permeability in governing local drug distribution within the FRT. The experimentally derived parameters provide quantitative inputs for FRT PBPK model development, and are expected to inform design of safe and effective localized therapies for women’s reproductive health.

## 1. Introduction

The female reproductive tract (FRT) represents a complex biological system with distinct anatomy regions, including the vagina, cervix, uterus, fallopian tubes, and ovaries, each characterized by dynamic physiological and structural properties [1]. These regional differences influence drug distribution and pose unique challenges for drug development and drug delivery to the FRT targeting sexually transmitted infections, gynecological diseases, and the prevention of unintended pregnancies [1,2]. Despite increasing interest in localized therapies for the FRT, the impact of biological factors on drug distribution across different biological fluids and tissue compartments within this space remains incompletely characterized, particularly with respect to solubility, matrix-specific binding, and permeability barriers. Understanding the physiological and drug-specific factors that govern biodistribution within the FRT is critical to predict local drug exposure and support the development of safe and effective therapeutic modalities delivering drugs directly to the FRT.

Drug distribution within the FRT is governed by multiple interconnected factors, among which drug solubility, matrix-specific binding, and membrane permeability are particularly crucial. A drug’s solubility in a specific biological fluid determines the extent to which a drug is available for transport [3]. In addition, matrix-specific binding in biological fluids and FRT tissues, defined here as the binding to proteins and all other components within a given biological matrix, including albumin, sex hormone-binding globulin, and tissue-specific structural proteins, can alter the unbound fraction of drug available for diffusion and pharmacological activity [4]. Specifically, the bioavailability and distribution of a drug and/or its active metabolite(s) are affected by the protein concentrations and protein types in the biological fluids and tissues [5]. Variability in protein expression in biological fluids and FRT tissues, as well as between individuals, may lead to differences in local drug exposure that cannot be captured by plasma pharmacokinetics alone. Membrane permeability across biological barriers also plays a major role in drug distribution. As different regions within the FRT display distinct anatomical architecture, epithelial thickness, and tight junction composition, it is crucial to understand how drugs permeate across different FRT tissue types [6]. Specifically, tissues in the lower regions of the FRT including the vagina and ectocervix contain multilayered stratified squamous epithelial cells that lack strong tight junctions [1,7]. On the other hand, tissues in the upper regions of the FRT including the uterus and fallopian tubes contain single-layer columnar epithelial cells with strong tight junction expressions [8,9]. Despite their importance, these parameters are not well characterized across the diverse compartments of the FRT.

Drug physicochemical properties further modulate these processes. Lipophilicity and ionization behavior of a given drug influence not only the solubility, but also matrix-specific binding interactions and membrane permeability. The extent of drug ionization at physiological pH influences its apparent solubility in biological fluids, electrostatic interactions with proteins, and ability to traverse epithelial barriers via passive diffusion. This is particularly relevant in the FRT, where pH varies across compartments (e.g., cervicovaginal fluid versus tissue interstitium), potentially altering local drug ionization and distribution. Importantly, these physicochemical factors also act in concert with tissue architecture and transporter mechanisms that determine the drug’s distribution into fluids and tissue compartments [10].

Although prior studies have examined individual aspects of drug disposition in the FRT, a critical gap remains in the availability of quantitative, integrated data describing solubility, matrix-specific binding, and permeability across multiple FRT compartments. This lack of comprehensive, matrix-specific datasets limits the development and parameterization of physiologically based pharmacokinetic (PBPK) models, which rely on experimentally derived inputs to predict local drug exposure. Furthermore, existing studies often evaluate isolated systems, making it difficult to systematically compare these parameters across biological matrices and tissue types. To address this gap, the present study systematically integrates in vitro and ex vivo approaches to quantify key determinants of drug disposition across the FRT. In this study, the solubility in plasma, matrix-specific binding in different biological fluids and FRT tissues, and membrane permeability of four model drugs were characterized to explore how these biological factors affect drug biodistribution in the FRT (Table 1) [11,12,13,14,15,16,17,18,19]. These model drugs were selected because they possess different physiochemical properties (e.g., lipophilicity, ionization) and drug transporter profiles, which allow us to systematically evaluate how diverse physicochemical and biological features influence drug distribution in the FRT. This selection enables mechanistic evaluation of how drug-specific properties influence solubility, binding, and permeability in biologically relevant matrices. In addition, these model drugs represent clinically relevant antiviral and contraceptive agents that are currently under development or use in intravaginal or intrauterine delivery.

The combined use of cell-based models and ex vivo human tissues allows complementary evaluation of drug transport processes. In vitro models provide controlled systems for assessing passive and transporter-mediated permeability, while ex vivo tissues preserve native architecture and barrier properties, enabling more physiologically relevant assessment of drug transport across the FRT. Integrating these approaches enables direct comparison across model systems, evaluation of suitability of in vitro models as a surrogate for tissue permeability assessments, and translation of experimental data into PBPK models. Specifically, this study aims to (1) quantify solubility in plasma, (2) characterize matrix-specific binding in plasma, cervicovaginal fluid, and FRT tissues, and (3) evaluate permeability across multiple FRT tissue types using complementary models. By generating quantitative, matrix-specific parameters within a unified experimental framework, this work provides critical inputs for PBPK model development and advances mechanistic understanding of drug disposition in the FRT. Most of the findings reported in this manuscript were included in a published abstract presented at the 2025 American Society for Clinical Pharmacology and Therapeutics Annual Meeting [20].

## 2. Materials and Methods

This study systematically evaluated key determinants of drug disposition within the female reproductive tract (FRT) using an integrated experimental approach. Plasma solubility, matrix-specific binding in biological fluids and tissues, and ex vivo permeability across FRT tissues were assessed. In vitro permeability studies using Caco-2 and 3D vaginal models provided additional mechanistic insight into passive and transporter-mediated transport. These complementary datasets were integrated to understand how physicochemical and biological factors influence drug distribution within the FRT, and to inform physiologically based pharmacokinetic (PBPK) model development.

### 2.1. Materials

DPV (CAS number-244767-67-7) and deuterated DPV (d4-DPV) were kindly provided by Population Council (New York City, NY, USA). Micronized LNG was purchased from Sterling Chemical Malta (Birzebbugia, Malta). Deuterated LNG (d6-LNG) was purchased from Cayman Chemical Company (Ann Arbor, MI, USA). MK-2048 and deuterated MK-2048 (d6-MK-2048) with >99% purity were kindly provided by Merck & Co. (Kenilworth, NJ, USA). EFdA and an isotopic internal standard, 13C15N3-EFdA, were custom synthesized at WuXi^®^ AppTec (EFdA) in Shanghai, China. Lucifer yellow was obtained from Sigma Aldrich (St. Louis, MO, USA). Fetal bovine serum (FBS), 100× penicillin-streptomycin-glutamine (pen-strep), Hank’s balanced salt solutions (HBSS) with and without Ca^2+^ and Mg^2+^, and dimethyl sulfoxide (DMSO) were obtained from Thermo Fisher Scientific (Waltham, MA, USA). The 0.25% trypsin and 0.1% ethylenediaminetetraacetic acid in HBSS (trypsin-EDTA), Dulbecco’s Modified Eagle Medium (DMEM), minimum essential medium, 10× phosphate-buffered saline (PBS), and Dulbecco’s phosphate-buffered saline (DPBS) were obtained from Corning Inc. (Corning, NY, USA).

Freshly excised human ectocervical, uterine (endometrial and myometrial), and fallopian tube tissue samples were collected from premenopausal women (age ranges 20–45, *n* = 3–4 donors per tissue type) undergoing hysterectomy through the Pitt Biospecimen Core at the University of Pittsburgh under expedited approved IRB protocol (ICR18110140-004, approved 2 November 2021). Detailed donor demographic and clinical characteristics, including menstrual cycle phase, and hormonal status, were not available due to the use of de-identified specimens. All tissues were de-identified and collected through an honest broker. Only macroscopically healthy regions of tissue, as determined by clinical evaluation at the time of surgery, were used for this study. Immediately upon collection, tissue samples were immersed in ice-cold Dulbecco’s Modified Eagle Medium (DMEM) and then transferred from the surgery site (Magee-Womens Hospital) to the laboratory (Magee-Womens Research Institute). Tissue samples were snap frozen and stored at −80 °C. DMEM and 10× phosphate-buffered saline (PBS), pH 7.4, were obtained from Corning Inc. (Corning, NY, USA). Pooled human plasma (K2 EDTA pooled gender) was purchased from BioIVT Inc. (Westbury, NY, USA). Rapid equilibrium dialysis (RED) device single-use inserts, reusable Teflon base, and hand-held homogenizer saw tooth probe 5–10 mm diameter were purchased from Thermo Fisher Scientific (Rockford, IL, USA). Analytical grade formic acid, acetonitrile, methanol, isopropyl alcohol, and water were obtained from Fisher Scientific (Waltham, MA, USA).

### 2.2. Ultra-Performance Liquid Chromatography Tandem Mass Spectrometry (LC-MS/MS)

The LC-MS/MS methods were adopted from the previously published work [15,18,21]. LC-MS/MS analysis of all samples was performed using a Waters Acquity UPLC coupled with Waters Xevo TQ-S (MassLynx v4.2 SCN 986, Waters Corporation, Milford, MA, USA) mass spectrometer equipped with an electrospray ionization source. Multiple reaction monitoring (MRM) was used for drug quantification in the positive ionization mode. The LC-MS/MS conditions, including the column type, internal standard, mobile phase, flow rate, MRM transitions, and total run time for each model drug and its internal standard, are summarized in Table S1. The LC-MS/MS methods were validated as per the recommendation of USFDA guideline [22]. Carryover was assessed by injecting a blank sample following the highest calibration standard, and no significant analyte signal was detected in the blank. Stable isotope-labeled internal standards were used to account for variability in extraction efficiency and potential matrix effects. The other performance metrics are summarized in Table S2.

### 2.3. Solubility of Model Drugs in Pooled Human Plasma

The solubility of model drugs was determined by dissolving 0.6–0.7 mg of drug in 0.5 mL of human pooled plasma in a silanized glass vial. For each model drug, the solubility study was performed in triplicates. The mixture was incubated on an orbital shaker at 500 rpm, 37 °C for 24 h. At the end of 24 h, each sample was transferred to a low-retention centrifuge tube and centrifuged at 3000 rpm for 10 min. The supernatant was filtered through a 0.2 μm PTFE filter, and 250 μL of filtered supernatant was mixed with 1 mL of acetonitrile in a microcentrifuge tube. After brief vortexing, the mixture was centrifuged at 3000 rpm for 10 min. The collected supernatant was analyzed by LC-MS/MS.

### 2.4. Matrix-Specific Binding of Model Drugs in Pooled Human Plasma, Human Cervicovaginal Fluid, and Human Excised FRT Tissues

Frozen excised human FRT tissues were thawed in a water bath set at 37 °C. Tissue samples were homogenized in Dulbecco’s PBS (without Ca^2+^ or Mg^2+^) with a 10-fold dilution using a hand-held homogenizer. The bicinchoninic acid (BCA) assay was performed using Pierce^TM^ BCA protein assay kits (Thermo Fisher Scientific (Rockford, IL, USA)) to quantify total protein concentration in the tissue homogenates. Tissue homogenate samples were stored at −80 °C until analysis. Human cervicovaginal fluid was collected between October 2023 and December 2024 by following the IRB protocol obtained by Dr. Sharon Hillier’s laboratory (IRB# STUDY20040351, approved 8 July 2020). In brief, participants were recruited at Magee-Womens Hospital and asked to wear a menstrual cup for 6–8 h. The menstrual cup was collected and transported to the laboratory in a 50 mL conical tube. Upon arrival at the laboratory, fluid in the conical tube was spun down at 1500 rpm for 15 min and stored at −80 °C until use for protein binding assessment.

In this study, matrix-specific binding refers to apparent binding, which reflects the combined effects of protein binding, lipid partitioning, and nonspecific interactions within biological matrices rather than discrete protein binding alone. Matrix-specific binding of DPV, MK-2048, EFdA, and LNG in human biological fluids (plasma, cervicovaginal fluid) and FRT tissue homogenates was determined by a rapid equilibrium dialysis protein binding assay as previously described [23]. Stock solutions for each model drug and its respective internal standard were prepared in DMSO, and 1× PBS was prepared and warmed at 37 °C. The protein binding assay of each model drug was set up in a similar fashion for all matrices. Specifically, 1 μM of each model drug was spiked into each matrix in triplicates. A concentration of 1 μM was selected to ensure measurable binding while remaining within a range where nonspecific saturation effects are minimized. A volume of 200 μL of each spiked sample was added to the donor chamber, and 350 μL of warm 1× PBS was added to the receptor chamber of the RED inserts. The reusable Teflon base was sealed and incubated at 37 °C for 6 h at 250 rpm on an orbital shaker.

The 6 h incubation period was selected based on previously validated RED protocols demonstrating equilibrium across compounds with diverse physicochemical properties. Although equilibrium was not independently confirmed for each compound in this study, prior validation of this method supports that equilibrium is achieved under these conditions [23]. After the incubation period, the samples were collected and stored at −20 °C before sample processing via liquid–liquid extraction (no more than 24 h to minimize potential analyte degradation). Samples were processed using liquid–liquid extraction with methyl tert-butyl ether (MTBE). Extracts were evaporated and reconstituted in mobile phase prior to LC-MS/MS analysis. Detailed extraction procedures are provided in the Supplementary Materials. Fraction unbound to biological fluids (f_u,fluid_) and apparent fraction unbound to FRT tissues (f_u,app_) were calculated using Equation (1). Fraction unbound to FRT tissues (f_u,tissue_) was determined using Equation (2) to take into account the dilution factor (D) of the tissue homogenates [24]. The % binding and % recovery were calculated using Equations (3) and (4), respectively.

(1) $$f_{u,\mathrm{fluid}}\;\mathrm{or}\;f_{u,\mathrm{app}}=1-\;(\frac{\mathrm{Donor}\;\mathrm{Area}\;\mathrm{Ratio}-\;\mathrm{Receiver}\;\mathrm{Area}\;\mathrm{Ratio}}{\mathrm{Donor}\;\mathrm{Area}\;\mathrm{Ratio}})$$

(2) $$f_{u,tissue}=\frac{\frac{1}{D}}{\left(\frac{1}{f_{u,app}\;}-1+\frac{1}{D}\right)}$$

(3) $$\%\;\mathrm{binding}\;=(1-f_{u,\mathrm{fluid}})\times 100\%\;\mathrm{or}\;(1-f_{u,\mathrm{tissue}})\times 100\%$$

(4) $$\%\;\mathrm{Recovery}=\frac{\left(C_{d,6h}\times \;V_{d,6h})+(C_{r,6h}\;\times \;V_{r,6h}\right)}{\left(C_{d,0h}\;\times \;V_{d,0h}\right)}\;\times \;100\%$$

### 2.5. Ex Vivo Human Tissue Permeability Studies

#### 2.5.1. Tissue Permeability

Bidirectional permeability studies for all model drugs were performed with human ectocervical, endometrial, and fallopian tube tissues using an Ussing Chamber system (Physiologic Instruments, Inc., San Diego, CA, USA) as previously described [19,25]. For ectocervical and endometrial tissues, a Stadie-Riggs tissue slicer (Thomas Scientific, Swedesboro, NJ, USA) was used to separate the epithelium from the stroma layer. For fallopian tube tissues, an Integra^®^ Miltex sterile scalpel (Integra Lifesciences, Princeton, NJ, USA) was used to cut longitudinally to open the tubes. Permeability across myometrial tissues was also performed, but not bidirectional due to their lack of epithelium. A Stadie-Riggs tissue slicer (Braintree Scientific, Braintree, MA, USA) was used to cut the myometrial tissue into thinner slices (not bidirectional). Human tissue thickness was measured using a digital micrometer caliper. The tissue integrity was evaluated by monitoring the TEER changes. The processed ectocervical, endometrial, myometrial, and fallopian tube tissues were mounted onto the 0.031 cm^2^ chamber slider. A small piece of tissue from each tissue sample was fixed in 10% formalin as the pre-treatment control for qualitative histology evaluation.

For DPV and LNG, Krebs–Ringer bicarbonate buffer (KRB) was used to perform tissue permeability assays. KRB was prepared using a recipe from the manufacturer (6.72 g/L sodium chloride, 0.42 g/L monobasic potassium phosphate, 0.054 g/L dibasic potassium phosphate, 2.10 g/L sodium bicarbonate, 0.18 g/L calcium chloride, and 0.11 g/L magnesium chloride). KRB was bubbled with carbogen (95% O_2_ and 5% CO_2_) for 1 h and then adjusted to pH 7.4 with 0.1 N sodium hydroxide. For MK-2048 and EFdA, HBSS was used to perform tissue permeability assays. A volume of 3 mL of KRB or HBSS was used in each chamber and carbogen was supplied throughout the experiment. Table S3 lists the stock and diluted concentrations used in the experiment. All permeability assays were conducted in KRB adjusted to pH 7.4 or HBSS to ensure consistent ionization conditions across experiments. This pH is close to physiological tissue pH and maintains the predominant ionization state for all four compounds under study. Although luminal FRT environments such as cervicovaginal fluid may exhibit lower pH, underlying tissue environments remain near neutral; therefore, pH 7.4 was selected to preserve tissue integrity and allow consistent comparison across compounds. Different transport media (KRB for DPV and LNG, and HBSS for MK-2048 and EFdA) were selected based on compound-specific compatibility and established assay conditions. Each compound was evaluated under consistent conditions, enabling valid within-compound comparisons.

For the apical to basolateral direction, each model drug solution was added in the apical side, and the samples were collected from the basolateral side. At predetermined time points (15, 30, 45, 60, 75, 90, 105, 120, 150, 180, 210, 240, 270, and 300 min), 1 mL samples were collected and the basolateral side was immediately replaced with 1 mL blank KRB or HBSS. Samples were analyzed using the validated LC-MS/MS method within 1 week from collection to minimize potential analyte degradation. At the end of each experiment, post-exposure tissue samples were collected and processed for histological evaluation as described in Section 2.5.2. The obtained TEER values were normalized against initial measurements at time 0.

The apparent permeability coefficient (P_app_) was calculated using Equation (5).

(5) P_app_ = (dQ/dt)/(A × C_0_)

where dQ/dt was the initial linear flux, the slope obtained from the linear regression plot between Q (cumulative amount transported to the receptor side) and t (time), A was the surface area of exposure (tissue surface area is 0.031 cm^2^, surface area of the Transwell^®^ used for Caco-2 cell permeability is 1.12 cm^2^, VK2/BJ surface area of the Transwell^®^ used for VK2/BJ cell permeability is 0.332 cm^2^), and C_0_ was the initial drug concentration at the donor side.

#### 2.5.2. Histology

Gross morphology of tissue samples was evaluated using hematoxylin and eosin (H&E) staining [19]. Tissue samples were fixed in 10% formalin, dehydrated in alcohol, cleared in xylene, and then embedded in paraffin wax. Five-micron sections of each embedded tissue were used to perform H&E staining following the manufacturer’s procedures (hematoxylin for 5 min, bluing reagent for 15 s, and eosin for 2 min) and mounted onto microscope slides using Cytoseal 60. Images under 10× or 20× magnification were acquired using the AxioCam software, version 4.9.1 (Carl Zeiss, San Diego, CA, USA) paired with the microscope, Axioskop 40 (Carl Zeiss, San Diego, CA, USA). Qualitative assessment of pre- and post-exposure tissue sample images was conducted to identify overt drug exposure-related changes in tissue morphology and barrier integrity using predefined morphological criteria, including epithelial integrity, tissue architecture, and evidence of degeneration or necrosis.

### 2.6. In Vitro Permeability

#### 2.6.1. Cell Culture

Human colon epithelial cells (Caco-2, ATCC HTB-37), human vaginal epithelial cells (VK2/E6E7, ATCC CRL-2616), and human BJ fibroblast cells (ATCC CRL-2522) were obtained from ATCC (Manassas, VA, USA). Caco-2 cells were cultured in ATCC formulated Eagle’s Minimum Essential Medium supplemented with 20% FBS, 1% L-glutamine, and 1% penicillin-streptomycin. VK2/E6E7 cells were cultured in Keratinocyte-Serum Free medium supplemented with 0.1 ng/mL human recombinant EGF, 0.05 mg/mL bovine pituitary extract, calcium chloride 44.1 mg/L, 1% L-glutamine, and 1% penicillin-streptomycin. BJ cells were cultured in ATCC formulated Eagle’s Minimum Essential Medium supplemented with 10% FBS, 1% L-glutamine, and 1% penicillin-streptomycin. Both Caco-2 and VK2/BJ (3D vaginal model) cell lines were cultured at 37 °C with 5% CO_2_ under fully humidified conditions. The cells were allowed to grow to 80–90% confluence and harvested by trypsinization using a solution containing 0.25% trypsin and 0.02% EDTA.

#### 2.6.2. Cell Permeability Studies

Caco-2 and 3D vaginal epithelial (VK2/BJ) models were established following previously described protocols with minor modifications (see Supplementary Methods) [26,27]. The quality of the monolayers and 3D model grown on the permeable membrane was assessed by measuring the transepithelial electrical resistance (TEER) of the monolayers using a Millicell-ERS (electrical resistance system) apparatus (Millipore, Bedford, MA, USA). Only Caco-2 monolayers displaying TEER values > 600 Ωcm^2^ and 3D model with TEER values > 50 Ωcm^2^ were used in the cell permeability studies.

Before each cell permeability study, cells were washed twice with HBSS and then incubated at 37 °C for 20 min with HBSS, followed by incubation at room temperature for 10 min and then the TEER values were measured. Permeability studies were conducted in a Transwell^®^ system. The drug concentrations used in these studies were 5 μM, 1.5 μM, 42.6 μM, and 16 μM for DPV, MK-2048, EFdA, and LNG, respectively (Table S3). The drug concentrations used in these studies were selected to ensure measurable transport across intact monolayers under sink conditions while maintaining barrier integrity. Concentrations were tailored to each compound based on its solubility, binding properties, and expected permeability to allow reliable quantification of flux within the experimental timeframe. For Caco-2 studies, the apical chamber, basal chamber, and sampling volumes were 0.5 mL, 1.5 mL, and 0.12 mL, respectively. For VK2/BJ studies, the apical chamber, basal chamber, and sampling volumes were 0.2 mL, 0.5 mL, and 0.05 mL, respectively. Cell permeability studies were conducted at 37 °C for 1.5 h with gentle shaking (50 rpm) and samples were collected from the receiver compartment at 0, 15, 30, 45, 60, and 90 min and from the donor compartment at 0 and 90 min. TEER values were measured at the end of each experiment to monitor the change in integrity of monolayers. HBSS-containing 300 µM Lucifer yellow (as a control) was added to the apical side of the Transwell^®^ and incubated in an orbital shaker at 37 °C for 1 h. A volume of 200 µL of HBSS from the basolateral chamber was transferred to a black 96-well plate and measured using a spectrophotometer SpectraMax M3 plate reader (Molecular Devices, San Jose, CA, USA) to determine the fluorescent absorbance using 485 nm excitation and 535 nm emission wavelengths. Drugs in all samples were analyzed using LC-MS/MS within 1 week to minimize potential analyte degradation. The apparent permeability coefficient (P_app_) was calculated using Equation (5) above. Efflux ratios were calculated as the ratio of B to A to A to B permeability. An efflux ratio > 2 was used as a threshold to indicate potential involvement of active efflux processes, based on commonly accepted criteria in permeability studies. This threshold is widely used to screen for transporter-mediated efflux, although it does not provide definitive evidence of transporter activity [28].

### 2.7. Statistical Analysis

Data are presented as mean ± standard deviation unless otherwise specified. Fraction unbound and apparent permeability values were log10-transformed prior to analysis. For binding analysis, we used Brown–Forsythe and Welch one-way ANOVA test with Games–Howell post hoc comparisons. For directional permeability comparisons (A → B vs. B → A), we used Welch’s unpaired *t*-test on log10(P_app_) for each tissue type and two-way ANOVA on log10(P_app_) for cell models using GraphPad Prism software version 10.4.1. For between-tissue comparisons, each direction (A → B and B → A) was analyzed separately using Brown–Forsythe and Welch’s one-way ANOVA on log10(P_app_) with Games–Howell post hoc comparisons. Data include both biological and technical replicates where applicable, and variability reflects inter-donor and experimental variation. All statistical analyses were performed using GraphPad Prism software version 10.4.1. A *p*-value < 0.05 was considered statistically significant.

## 3. Results

### 3.1. Solubility of Model Drugs in Human Pooled Plasma

Though aqueous solubility is often reported in the literature, plasma solubility is rarely reported. However, the presence of proteins is known to affect solubility, especially for hydrophobic compounds [29]. In this work, human plasma solubility was determined for DPV, LNG, MK-2048, and EFdA following 24 h of incubation of excess amount of each drug in plasma. We observed substantial differences in solubility among the four model drugs (Table S4). As expected, the solubility of model drugs in human pooled plasma followed the same trend of hydrophobicity, where the most hydrophobic drug, DPV, has the lowest solubility and the most hydrophilic drug, EFdA, exhibited markedly high solubility. The plasma solubility values observed in this work were significantly higher (~2× to >30×) than the corresponding aqueous solubility previously reported (Table S4), confirming the enhancement effect of proteins on drug solubility [29]. In addition, this trend is also regulated by ionization behavior, as the model drugs that remain largely unionized (DPV and LNG) at physiological pH exhibit lower apparent solubility compared to more polar or partially ionized compounds such as MK-2048 and EFdA.

### 3.2. Matrix-Specific Binding of Model Drugs in Human Biological Fluids and Tissues

Overall, clear differences in the extent of matrix-specific binding were observed among the four model drugs across all biological matrices (Table 2). DPV was found to have the highest extent of apparent binding (97.49–99.45%) with no significant difference between pooled human plasma, human cervicovaginal fluid, and human FRT tissue homogenates. LNG and MK-2048 also demonstrated high apparent binding with matrix-dependent variability. Specifically, LNG exhibited significantly higher binding in plasma and FRT tissue homogenates (92–98%) compared to cervicovaginal fluid (89.6 ± 1.31%, *p* < 0.0001). In contrast, MK-2048 showed significantly higher binding in plasma compared to cervicovaginal fluid (96.1 ± 0.33% vs. 84.8 ± 3.0%, *p*-value < 0.001), while binding in FRT tissue homogenates (97.9–99.1%) was numerically higher but not statistically different from cervicovaginal fluid. Unlike the other compounds, EFdA was the least matrix-bound compound and highest variability in pooled human plasma, human cervicovaginal fluid, and human FRT tissues. For EFdA, we observed a numerically higher apparent binding in human FRT tissues compared to pooled human plasma and human cervicovaginal fluid, although these differences did not reach statistical significance. Recovery across all matrices ranged from 81 to 105% and showed no systematic differences between plasma, cervicovaginal fluid, and tissue homogenates. These findings indicate consistent extraction efficiency across matrices.

### 3.3. Tissue Permeability and Histology

**Figure 1:**
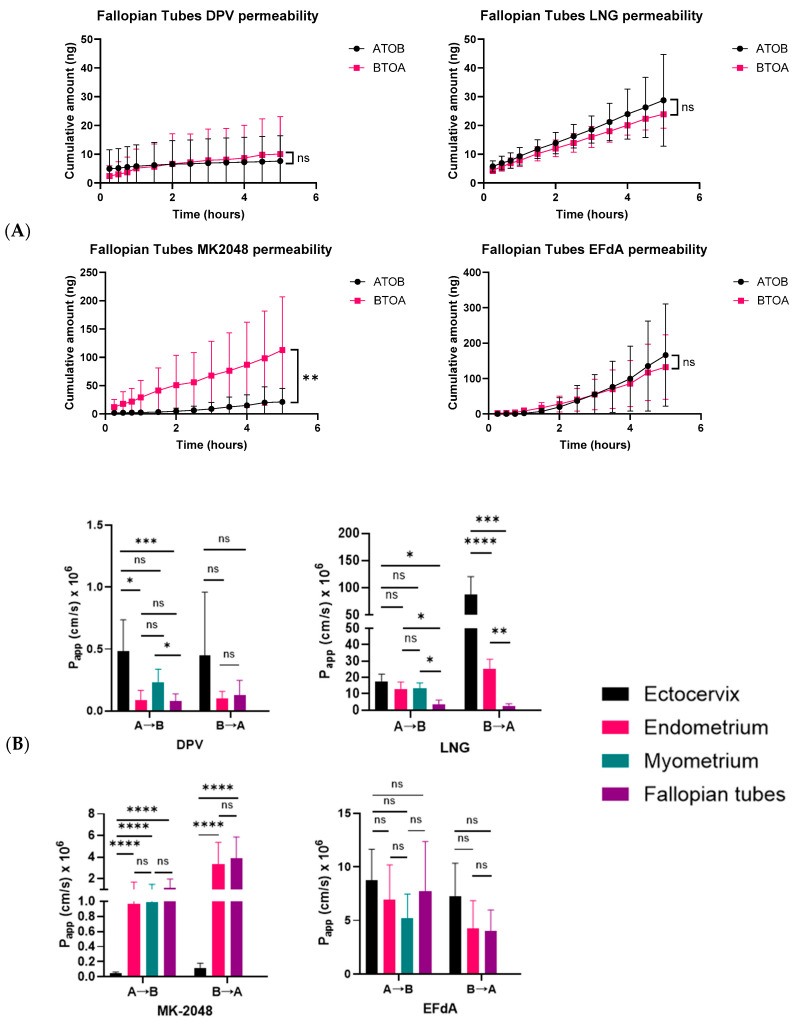
(**A**) Representative bidirectional transport of model drugs across human fallopian tubes. Data are shown as the mean ± standard deviation (*n* = 6–12 replicates). Statistical differences determined by Welch’s unpaired *t*-test on log10(P_app_). (**B**) Comparison of apparent permeability for each direction across all tissues for each model drug. Data presented as mean ± standard deviation. Statistical differences determined by Brown–Forsythe and Welch’s one-way ANOVA on log10(P_app_) with Games–Howell post hoc comparisons with *p* < 0.05 considered significant. (* ≤0.05, ** <0.01, *** <0.001, **** <0.0001).

Regional and compound-dependent differences in permeability were observed across FRT tissues (Figure 1A,B). The linear transport of DPV, MK-2048, LNG, and EFdA across the human ectocervix, myometrium, endometrium, and fallopian tubes was plotted, and the corresponding slopes were used to calculate P_app_. As an example, Figure 1A shows linear transport of all model drugs across fallopian tubes. The P_app_ values and calculated efflux ratios for each model drug are listed in Table 3.

For DPV and EFdA, the B to A flux were not significantly higher than the A to B flux, which results in efflux ratios (0.52 to 1.6) less than 2 across ectocervix, endometrium, and fallopian tubes. For MK-2048, the B to A flux were higher than the A to B flux, which results in efflux ratios (2.55 to 3.53) greater than 2 across ectocervix, endometrium, and fallopian tubes. For LNG, the B to A flux were significantly higher than the A to B flux across ectocervix, which results in efflux ratios greater than 2. On the other hand, the B to A flux was similar or not significantly higher than the A to B flux across endometrium and fallopian tubes, which results in efflux ratios less than 2.

Regional differences in permeability across the ectocervix, endometrium, myometrium, and fallopian tubes are summarized in Figure 1B, which are consistent with the directional data shown in Table 3. Permeability data reflect measurements from multiple independent donors (biological replicates), with multiple technical replicates performed per donor and summarized as mean ± standard deviation in Table 3. For DPV, A to B flux was significantly higher in the ectocervix than in the endometrium and fallopian tubes (0.45 ± 0.38 × 10^−6^ cm/s vs. 0.087 ± 0.075 × 10^−6^ cm/s vs. 0.08 ± 0.09 × 10^−6^ cm/s, *p*-value < 0.05), whereas B to A flux across all FRT tissues were not significantly different. For LNG, ectocervix showed the highest permeability, with A to B flux being significantly greater than that compared to the fallopian tubes (17.7 ± 7.84 × 10^−6^ cm/s vs. 3.32 ± 3.10 × 10^−6^ cm/s, *p*-value < 0.05), while B to A flux demonstrated the same patterns (87.2 ± 60.4 × 10^−6^ cm/s vs. 2.62 ± 1.24 × 10^−6^ cm/s, *p*-value < 0.001). In addition, permeability in the endometrium was significantly higher than in the fallopian tubes for both A to B and B to A flux (A to B: 13 ± 8.15 × 10^−6^ cm/s vs. 3.32 ± 3.10 × 10^−6^ cm/s, *p*-value < 0.05; B to A: 25.4 ± 10.7 × 10^−6^ cm/s vs. 2.62 ± 1.24 × 10^−6^ cm/s, *p* < 0.01). Similarly, the myometrium exhibited higher A to B flux compared to the fallopian tubes (13.1 ± 7.07 × 10^−6^ cm/s vs. 3.32 ± 3.10 × 10^−6^ cm/s, *p* < 0.05). In contrast to the other three model drugs, the A to B and B to A flux of MK-2048 in the ectocervix were significantly lower compared to the upper FRT tissues (e.g., A to B: 0.047 ± 0.029 × 10^−6^ cm/s vs. 0.97 ± 0.85 × 10^−6^ cm/s, *p*-value < 0.0001; B to A: 0.12 ± 0.074 × 10^−6^ cm/s vs. 3.35 ± 2.19 × 10^−6^ cm/s *p*-value < 0.0001). For EFdA, A to B and B to A flux were broadly similar across tissues with no consistent significant pairwise differences.

**Figure 2:**
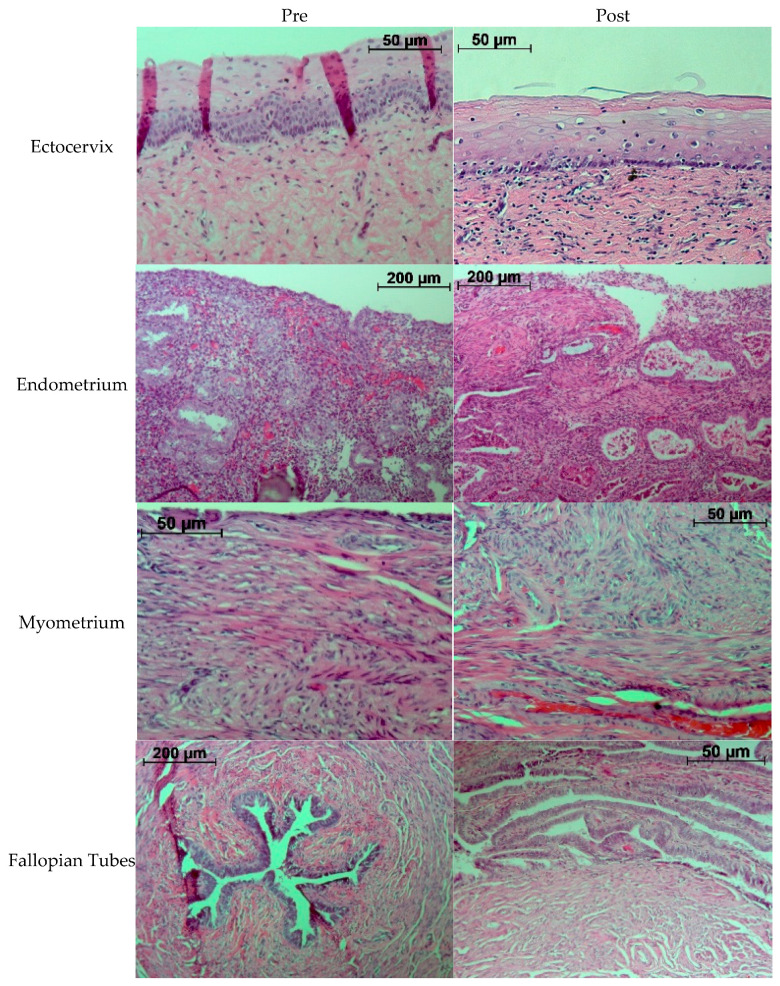
Representative histology of human ectocervix, endometrium, myometrium, and fallopian tubes before and after tissue permeability study using dapivirine (10×–20×).

Qualitative histological evaluation demonstrated that all tissue samples maintained morphology between pre- and post-exposure to the model drugs and there was no tissue degeneration or necrosis. Specifically, the tissue architecture like the stratified squamous epithelium and stroma of ectocervix, the endometrial glands and stroma, and the mucosal folds of fallopian tubes remained intact throughout the experiments. Representative histology images of human ectocervix, myometrium, endometrium, and fallopian tubes pre- and post-exposure to DPV are shown in Figure 2.

### 3.4. Cell Permeability

Caco-2 cells used for the generation of monolayers on Transwells were restricted within passage numbers 23 to 38. Cells higher than passage number 38 were not preferred for seeding since the increase in number of passages will result in unintentional selection of faster-growing subpopulations of cells expressing different subsets of characteristics, which will in turn affect the differentiation process and compromise the integrity of the monolayer [31]. Similarly for the 3D vaginal model preparation, the VK2/E6E7 cells within passage numbers 53 to 68 were used.

**Figure 3:**
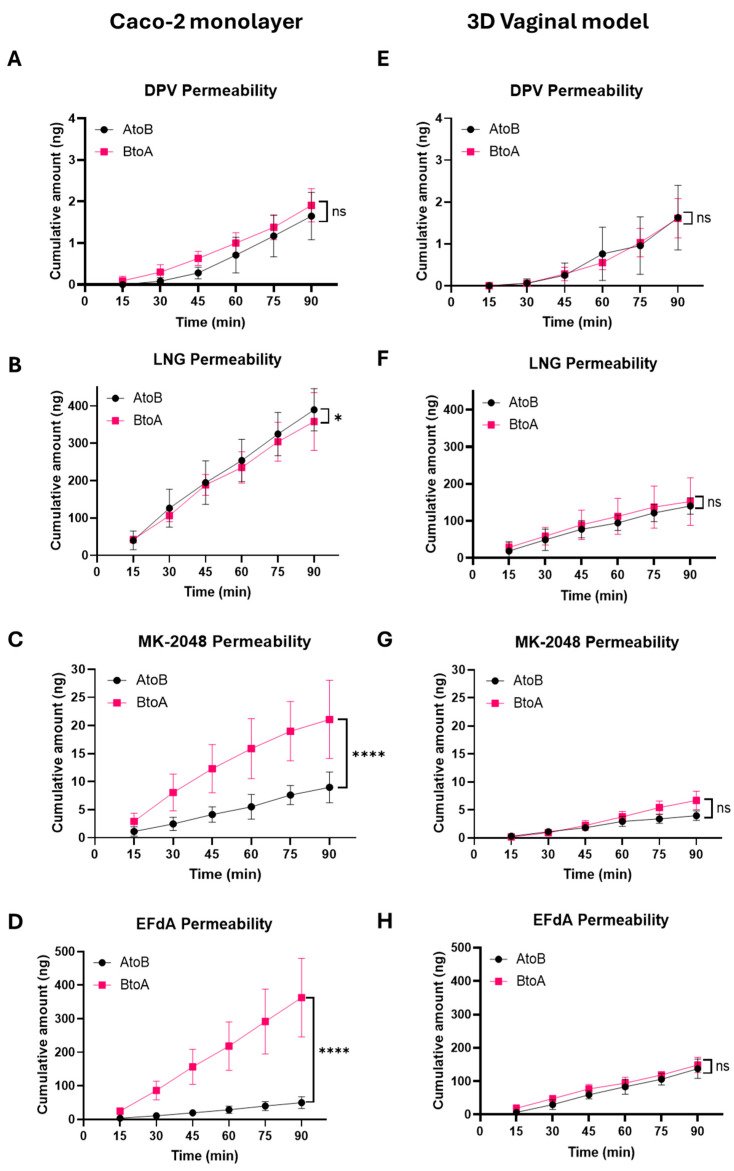
Bidirectional transport of model drugs across Caco-2 monolayer (**A**–**D**) and 3D vaginal model (**E**–**H**). Data presented as mean ± standard deviation from *n* = 9–15 (Caco-2) and *n* = 10–12 (3D vaginal model) independent replicates, as detailed in Table 4. Statistical differences determined by Brown–Forsythe and Welch’s one-way ANOVA on log10(P_app_) with Games–Howell post hoc comparisons with *p* < 0.05 considered significant. (* ≤0.05, **** <0.0001).

The linear transport of DPV, MK-2048, LNG, and EFdA across the Caco-2 monolayer and 3D vaginal models is shown in Figure 3. The P_app_ values and calculated efflux ratios for each model drug are listed in Table 4. Across the Caco-2 cell monolayer, the B to A flux of MK-2048 and EFdA was significantly higher than the A to B flux (MK-2048: 102 ± 75.8 × 10^−6^ cm/s vs. 39.3 ± 16.7 ×10^−6^ cm/s, *p*-value < 0.0001; EFdA: 6.13 ± 1.84 × 10^−6^ cm/s vs. 0.09 ± 0.26 × 10^−6^ cm/s, *p*-value < 0.0001), which resulted in an efflux ratio above 2 (Table 4). On the other hand, the B to A flux was not significantly higher than the A to B flux for DPV and LNG, which resulted in an efflux ratio below 2. In comparison, the efflux ratio values of all four model drugs were found to be below 2 in the 3D vaginal model (Table 4). TEER values before and after each experiment were measured to be 798.02 ± 133.71 and 687.49 ± 110.32 Ωcm^2^ in Caco-2 cell monolayer and 73.51 ± 11.29 and 77.58 ± 15.45 Ωcm^2^ in 3D vaginal model, respectively. There were no significant differences between (*p*-value > 0.05). before and after each experiment, which indicated that the quality of the cell monolayers and 3D vaginal models was maintained throughout the experiment. Lucifer yellow barrier assay showed <2% pass through, which indicated that the in vitro cell models were intact throughout the experiment.

## 4. Discussion

Solubility, matrix-specific binding, and permeability are key determinants of a drug’s biodistribution and pharmacokinetic and pharmacodynamic profile, which makes it essential to understand these properties across biological fluids and tissues within the FRT to optimize intravaginally administered therapies. Therefore, the goal of this study is to determine how different biological factors within the FRT potentially affect the biodistribution of therapeutic agents with different physicochemical properties.

In vitro-derived parameters are critical for the development of PBPK models of the FRT, as they define drug distribution, retention, and transport within this highly compartmentalized system [32]. Solubility in biological fluids determines the ability of the drug to be readily transported and achieve required pharmacological response at the target sites. Apparent binding to plasma and FRT tissues (e.g., cervix, endometrium, myometrium, fallopian tubes) governs the unbound fraction of a drug available for tissue penetration and pharmacological activity, while binding within cervicovaginal fluid influences luminal drug persistence and local exposure. Tissue-specific binding is particularly important in the FRT, where variations in lipid content, extracellular matrix composition, and hormonal regulation can lead to marked differences in drug partition relative to plasma [13]. Cervicovaginal fluid differs fundamentally from plasma and reproductive tract tissues in both origin and composition. As a mucosal secretion with low classical protein content and a mucin-rich matrix dominated by glycoproteins, lipids, and cellular debris, cervicovaginal fluid exhibits drug binding behavior that cannot be inferred from plasma or tissue measurements alone. Permeability data generated from epithelial cell models such as Caco-2 provide controlled and reproducible mechanistic insight into passive and transporter-mediated drug flux across mucosal barriers, supporting parameterization of epithelial transport processes and assist in vitro–in vivo extrapolation (IVIVE) of absorption processes [33]. Complementary ex vivo permeability studies using human cervical, uterine, and fallopian tube tissues capture the integrated effects of tissue architecture and tight junctions, enabling more physiologically relevant estimates of drug absorption and intercompartmental transfer. Comparing permeability outcomes across these in vitro and ex vivo platforms allows assessment of the extent to which in vitro models can recapitulate tissue-level transport processes and reliably parameterize PBPK models of the FRT, thereby supporting their use as practical alternatives when access to fresh FRT tissues is constrained. Together, these in vitro and ex vivo parameters reduce model uncertainty, improve prediction of local and systemic drug exposure, and enhance the translational value of PBPK models for evaluating drug disposition and efficacy in the FRT.

Only the unbound fraction of a therapeutic agent is available for distribution and metabolism, and therefore is expected to exhibit pharmacological activities [34]. Accordingly, these measurements provide insight into relative drug disposition across matrices. However, they should be interpreted as comparative indicators rather than direct predictors of in vivo exposure. The model drugs included in this study exhibit distinct lipophilicity, pKa, and solubility profiles, with DPV being the most lipophilic (logP = 5.6) and least soluble in plasma, and EFdA being the most hydrophilic (logP = −0.4) and most soluble in plasma. In addition to lipophilicity, ionization state is an important determinant of apparent solubility. Compounds that are predominantly ionized at physiological pH generally exhibit higher aqueous solubility, whereas neutral and highly lipophilic compounds tend to exhibit limited solubility. In the present study, both lipophilicity and ionization contributed to the observed solubility trends, with lipophilicity acting as the primary driver and ionization modulating solubility across matrices. However, the relative contributions of pKa and lipophilicity could not be quantitatively deconvoluted, and should therefore be interpreted as qualitative relationships. Although human cervicovaginal fluid exhibits a lower pH between 4 and 5, the ionization state of the four model drugs is largely unchanged within this range, and therefore, pH is not expected to be a major driver of the binding behavior of our model drugs. Instead, the lower apparent binding observed in cervicovaginal fluid (except EFdA) reflects its substantially lower protein content and the mucin-rich matrix. Specifically, DPV and MK-2048 may become slightly more protonated in cervicovaginal fluid, but the overall apparent binding patterns observed in our study reflect matrix-specific protein composition rather than pH-driven ionization effects. On the other hand, LNG and EFdA remain essentially non-ionized in this range of pH 4–5, which indicates that cervicovaginal fluid binding is regulated primarily by the reduced protein content and mucin-rich environment. EFdA showed higher apparent binding in cervicovaginal fluid compared to plasma, which is most likely due to its strong hydrophilicity and affinity for mucin-rich glycoproteins in cervicovaginal fluid. The viscoelastic, negatively charged mucin network can entrap or interact with EFdA, which results in a higher apparent binding in cervicovaginal fluid. The tissue binding of the model drugs also agrees with their relative hydrophobicity and pKa. Among the four model drugs, we observed the highest variability in apparent binding between different tissue types for LNG. Currently, there are limited data regarding whether these model drugs are primarily bound to albumin, alpha-1-acid glycoprotein, and/or other proteins. However, LNG has been reported to primarily bind to sex hormone-binding globulin (SHBG) [35]. Variability for LNG may be influenced by differences in SHBG levels across matrices and potential interactions with binding proteins. The levels of SHBG within tissues may be affected by several factors including age, sex, body fat, health conditions, lifestyle factors, and the use of hormone therapy [36]. Since the tissues used for matrix-specific binding measurements were sourced from different donors, underlying differences in SHBG levels cannot be excluded, which may partially explain the high variability observed in our tissue matrix-specific binding experiments for LNG. Prior PBPK modeling has demonstrated that incorporating SHBG-LNG binding dynamics is essential for accurately predicting LNG pharmacokinetic, including its distribution and drug–drug interactions. In particular, Cicali et al. developed a mechanistic PBPK model that explicitly modeled LNG’s binding and showed that SHBG levels are a major determinant of LNG exposure and interaction potential. Their findings support our observation of higher inter-tissue variability in LNG binding, which suggests that donor-specific SHBG abundance in FRT tissues may contribute to the heterogeneous binding behavior we observed across FRT tissues. This prior work underscores the need for future FRT-focused PBPK models to explicitly incorporate SHBG variability when simulating LNG or other steroid hormones [37]. EFdA also exhibited higher tissue binding compared to human cervicovaginal fluid and plasma. Unlike the other compounds evaluated, EFdA, a nucleoside analogue, undergoes rapid intracellular phosphorylation to its active triphosphate metabolites, which promotes cellular and tissue retention. EFdA is not extensively metabolized by hepatic CYP enzymes and is primarily cleared via renal pathways, with systemic disposition reflecting the parent compound [14]. As a result, the higher apparent tissue binding observed for EFdA likely reflects intracellular sequestration and association with cellular components rather than classical extracellular protein binding alone. This interpretation is consistent with clinical data that demonstrated high and sustained concentrations of islatravir-triphosphate in homogenized cervical, vaginal, and rectal tissues and isolated rectal cells following oral dosing [38]. For EFdA, the lower extent of binding may increase sensitivity to small experimental or matrix-related differences, resulting in greater relative variability. Although recovery values showed minor variability across matrices, all values were within an acceptable range (81–105%), indicating consistent extraction efficiency. No systematic matrix-dependent differences were observed, suggesting that analytical variability is unlikely to explain the observed differences in apparent binding. Therefore, the observed variability in binding is more likely attributable to biological factors such as matrix composition rather than assay-related effects.

Membrane permeability governs the extent and rate at which a compound moves from different biological fluids into target and non-target tissues. The concentrations used in tissue and cell permeability studies were selected to ensure quantifiable, linear transport across intact barriers under sink conditions. Higher donor concentrations were required in ex vivo tissue studies to maintain a stable concentration gradient and detect measurable flux, whereas lower, differentiated concentrations were used in in vitro models to preserve monolayer integrity and allow mechanistic evaluation of passive and transporter-mediated permeability. Although these concentrations exceed extracellular levels observed clinically, our studies were designed to characterize intrinsic transport properties relevant for interpreting tissue distribution and informing translational pharmacology rather than to directly mimic human. The pKa values of the four model drugs indicate that all compounds remain predominantly unionized at pH 7.4. Therefore, the permeability results reflect the physiologically relevant neutral species for each drug. Although cervicovaginal fluid exhibits an acidic pH 4–5, the underlying FRT tissues maintain physiological intracellular and interstitial pH values (~7.0–7.4). Therefore, permeability assays were performed using pH-neutral buffers to preserve tissue viability, maintain tight junction integrity, and capture intrinsic tissue permeability. For DPV, both A to B and B to A fluxes were numerically higher in the ectocervix relative to the upper FRT tissues. Given that DPV is not a known substrate for any drug transporters, one possible explanation for this trend is that the tight junction in the lower reproductive tract tissues is weaker than that in the upper reproductive tract tissues [19]. For MK-2048, the A to B and B to A flux was consistently lower in the ectocervix compared to the upper FRT tissues (endometrium, myometrium, and fallopian tubes). This pattern reflects both the limited passive permeability of MK-2048 across the stratified squamous epithelium of the ectocervix and the influence by P-gp and BCRP, for which MK-2048 is a known substrate [17,18]. The observed directional transport for MK-2048 is consistent with potential efflux transporter involvement. Supporting this interpretation, recent work from our group demonstrated expression and localization of key efflux transporters, including P-gp, BCRP, and MRP4, across FRT tissues with regional differences [39]. However, transporter expression was not directly measured for the tissues used in the permeability experiments described in this study, and therefore these findings should be interpreted as indirect evidence of transporter-mediated processes. In addition, efflux ratios alone do not provide definitive mechanistic evidence, and transporter activity was not directly evaluated in this study, though it was previously evaluated in MDCKII cells and ectocervical tissues [18,30]. For LNG, the A to B and B to A flux across the ectocervix, endometrium, and myometrium was significantly higher than that across fallopian tubes. The fallopian tube epithelium forms a structurally tighter barrier than cervical or endometrial epithelium due to its highly folded mucosa, specialized ciliated and secretory cells, and complex columnar epithelial architecture, which together reduce passive paracellular permeability [40,41]. In addition, previous findings have reported that LNG may result in changes in surface epithelium and local mediators regulating endometrial function [42], which may have contributed to the higher apparent permeability of LNG across the endometrium observed in our study. On the other hand, Maclean et al. reported that the fallopian tube epithelium exhibits distinct molecular and functional characteristics compared to the matched endometrium explants, including lower hormone responsiveness to estrogen, lower epithelial cell proliferation levels, and static steroid receptor activity regardless of the cycle phase [43]. Similarly, Li et al. reported no changes in the levels of fallopian tubal epithelial cell receptive markers when exposing tubal epithelial cell line OE-E6/E7 to contraceptive doses of LNG and progesterone [44]. For EFdA, the A to B and B to A flux was similar across all tissue types, which aligns with the tissue binding results and the low hydrophobicity of EFdA. In addition, we observed an efflux ratio of <2 across all tissue types with EFdA. Despite EFdA being a BCRP substrate in the Caco-2 model, this in vitro finding is not considered to be clinically significant, as EFdA at clinically relevant concentrations was neither a victim nor a perpetrator of any major drug transporters or drug-metabolizing enzymes involved in drug–drug interactions [14]. The observed permeability differences across tissues may reflect variations in epithelial structure, including differences in tissue thickness, cellular organization, and barrier properties such as tight junction integrity. These structural factors can influence the extent of drug diffusion; however, their specific contribution was not directly evaluated in this study and should be interpreted as a plausible contributing mechanism.

Our study also aimed to compare the permeability of the model drugs between the in vitro and ex vivo models to determine the potential of in vitro models as suitable surrogates when tissue accessibility is limited. Caco-2 cell monolayer was included, as this model is well-established and commonly used in pre-clinical screening of drug permeability and identification of potential substrates, inhibitors, and inducers of major transporters including P-gp, BCRP, and MRP4 [33]. The Caco-2 cell model is also recognized from an FDA regulatory perspective as a relevant in vitro system for early screening of drug transporter interactions, as it expresses key uptake and efflux transporters, and is recommended in FDA guidance for evaluating transporter-mediated drug–drug interaction potential [28]. Considering that the Caco-2 cell line is a human intestinal epithelial cell line derived from colorectal cancer, we also evaluated drug permeability in VK2/E6E7 cells, immortalized human vaginal epithelial cells which may be more relevant to the FRT tissues. Since it was observed that culturing epithelial cells in the absence of fibroblast cells and collagen on Transwell would result in reduced thickness of the 3D model, a thin layer of collagen was coated on the basal side of the Transwell, and BJ fibroblast cells were seeded on top of the collagen to form a 3D vaginal model. The incorporation of BJ fibroblast cells simulates the natural basement membrane environment of epithelial tissue [26]. Comparison of the permeability data obtained in these two cell models makes it apparent that DPV and LNG had efflux ratios of less than 2 regardless of the model used, while MK-2048 and EFdA showed significant differences between the two cell models: efflux ratios were higher than 2 in the Caco-2 cells, but lower than 2 in the VK2/E6E7 cells. These observations correlate with each model drug’s status as a substrate of efflux transporters and each cell model’s gene expression profile. We observed high expression levels of P-gp, BCRP, and MRP4 in Caco-2 cells, but lower expression of these transporters in VK2 cells, which agree with previous findings [45]. DPV and LNG are not known to be substrates of P-gp and/or BCRP transporters, and thus their efflux ratios are not greatly influenced by the gene expression profiles of the cell lines. In comparison, MK-2048 is a known substrate for P-gp and BCRP, and EFdA is a known substrate for BCRP, thus their efflux by the Caco-2 cells becomes more pronounced due to the Caco-2 cells’ high expression of P-gp and BCRP transporters [14,17,18]. On the other hand, VK2/E6E7 cells have lower expression levels of several efflux transporters, including P-gp, BCRP, and MRP4, thus it is reasonable that it would not be able to reflect the transporter-substrate status of MK-2048 and EFdA [14,17,18,45]. In addition to the efflux ratios, the apparent permeability of the model drugs for A to B and B to A flux was significantly higher in the in vitro models compared to the ex vivo models. The higher apparent permeability of the model drugs observed in the in vitro models is expected due to their simplified biological barriers lacking the tissue architecture and several physiological factors [46]. Despite the difference in the apparent permeability, the transporter-substrate status of the four model drugs was found to be largely consistent between the Caco-2 in vitro model and FRT tissue ex vivo models. The 3D vaginal model, on the other hand, failed to reflect the active efflux of MK-2048. We are in the process of developing another cell line model which may be more relevant to the FRT tissues and a better surrogate for tissue permeability studies.

Unlike prior studies that evaluate permeability in isolated systems, this study directly integrates solubility, matrix-specific binding, and permeability across multiple in vitro and ex vivo platforms within a unified framework. This enables a systematic comparison of drug disposition processes across biological scales, from simplified cell models to intact human tissues that provided insight into region-specific drug transport within the FRT. The interplay among solubility, matrix-specific binding, and tissue permeability was clearly demonstrated in the biodistribution of the model drugs evaluated in our study. Beyond lipophilicity, pKa provides a mechanistic basis for understanding this interplay. The extent of drug ionization at physiological pH determines the fraction of compound available in an unionized form capable of passive membrane diffusion, while also influencing apparent solubility and electrostatic interactions with biological matrices. Highly hydrophobic and largely unionized compounds, such as DPV, exhibited markedly reduced solubility and enhanced apparent binding, resulting in substantially lower tissue permeability (except for the cervical tissue). In contrast, more polar or partially ionized, highly hydrophilic compounds, such as EFdA, demonstrated significantly high solubility and reduced apparent binding, leading to substantially higher tissue permeability. This phenomenon emphasizes the fundamental understanding that only the unbound fraction that is available at the tissue membrane is able to diffuse across biological barriers. Beyond these three key parameters, drug transport mechanisms further influenced biodistribution, as observed for MK-2048. Given that MK-2048 is a known substrate to P-gp and BCRP efflux transporters, its transmembrane distribution is additionally influenced by the availability and interaction with P-gp and BCRP efflux transporters, which modulate its movement across the tissue membrane [17,18]. Overall, our study underscores the importance of comprehensive characterization of key physicochemical and biological parameters to obtain a comprehensive understanding of drug distribution. These findings highlight that optimal drug candidates for local FRT delivery require balanced physicochemical properties, including lipophilicity, controlled ionization, and matrix binding, and may represent optimal candidates for local FRT administration, as they provide sufficient solubility while maintaining the ability to penetrate tissue barriers and achieve sustained local exposure. The relative importance of these factors will depend on the desired therapeutic outcome, including retention within luminal fluids versus penetration into deeper tissue compartments.

There were some limitations to this study. First, our tissue, plasma, and cervicovaginal fluid samples used to measure matrix-specific binding of the model drugs were not from matched donors. A clinical study conducted to collect matched tissues, plasma, and cervicovaginal fluid samples may be necessary to minimize the inter- and intra-donor variability. For the current work, SHBG concentrations were not measured in individual plasma, cervicovaginal fluid, or tissue samples. Given that LNG binds extensively to SHBG, inter-individual variability in SHBG levels may contribute to the observed variability in LNG protein binding and tissue permeability. Within this future clinical study, relevant demographic and clinical data can be collected to account for confounding factors affecting protein levels, protein content, and SHBG concentrations. In addition, the measured matrix-specific binding represents apparent binding derived from tissue homogenates. Unlike pure protein binding, apparent binding reported in this manuscript reflects a composite of protein binding, lipid partitioning, and nonspecific interactions. Therefore, the reported values should be interpreted as overall drug–matrix association rather than discrete protein binding. This is particularly relevant for lipophilic compounds, where nonspecific partitioning may contribute substantially to apparent binding. It is noted that this study used tissue homogenates for matrix-specific binding assessment. While homogenization enables quantification of overall drug–matrix interactions, it does not fully preserve the spatial organization, cellular architecture, and microenvironmental heterogeneity of intact tissues. Therefore, these apparent binding results should be interpreted as estimates of overall matrix association rather than precise representations of in situ binding within native tissue environments. Next, VK2/E6E7 cells are considered to be relevant to the objective of our study, which is to assess different factors influencing biodistribution within the FRT. However, the lack of overexpression of key drug transporters including P-gp, BCRP, and MRP4 in this cell line might limit its use for permeability studies. An alternative in vitro model that closely recapitulates the physiological factors of the actual FRT tissues might be needed to assess and compare the permeability of the model drugs against the ex vivo model. We are currently developing a new in vitro model for permeability studies. Additionally, for hormonal drugs similar to LNG, further analyses beyond evaluation of tissue integrity (via TEER and H&E staining) are likely warranted to assess cellular changes and morphological modifications in cervical and endometrial tissues following prolonged drug exposure. Lastly, matrix effects were not formally assessed, and represent a limitation; however, internal standards and consistent recovery across matrices suggest minimal impact on quantification.

## 5. Conclusions

In this study, we systematically characterized solubility, matrix-specific binding, and membrane permeability of four model drugs with diverse physicochemical and transporter profiles across key compartments of the FRT using complementary in vitro and ex vivo approaches. Our findings provided matrix-specific estimates of apparent binding and regional permeability differences among human plasma, cervicovaginal fluid, and FRT tissues. These results highlighted the significant interplay between the dynamic anatomical and physiological characteristics of the FRT and local biodistribution of model drugs with distinct physiochemical properties and transporter-substrate status. Collectively, our work will enhance the understanding of drug disposition within the FRT. These experimentally derived solubility, matrix-specific binding, and tissue permeability data can be integrated into PBPK models of the FRT to improve predictions of local drug exposure and guide the development of safer and more effective intravaginal and intrauterine therapies.

## Figures and Tables

**Table 1:** Summary of relevant physicochemical properties of the model drugs [11,12,13,14,15,16,17,18,19]. NR = No report in the literature.

Model Drug	Dapivirine (DPV)	Levonorgestrel (LNG)	MK-2048	Islatravir (EFdA)
Structure	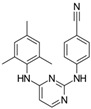	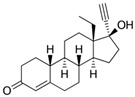	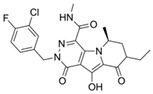	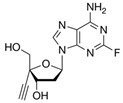
Route of administration	Intravaginal (vaginal ring)	Oral; subdermal implants; intrauterine (e.g., IUD)	Evaluated for intravaginal delivery	Investigational drug for oral and implant delivery
LogP	5.6	3.3	2.5	−0.42
pKa	5.3	Neutral in human body	9.79	<3
Molecular weight (g/mol)	329.4	312.4	461.9	293.25
Transporter profile	NR	NR	P-gp and BCRP substrate [17,18]	BCRP substrate [14]
Metabolic pathways	CYP1A1 and CYP3A4 enzyme substrate [19]	CYP3A4 enzyme substrate [11]	CYP1A1 enzyme substrate [18]	Adenosine deaminase [14]
Volume of distribution (L/kg)	NR	~1.8	~1.26	~0.5

**Table 2:** Matrix-specific binding of each model drug in different human biological fluids and human reproductive tract tissues. Data are shown as the mean ± standard deviation (*n* = 3–6 technical replicates). * = Data were obtained from Ref. [18]. Notes: Binding values represent apparent matrix association measured by rapid equilibrium dialysis.

Model Drug	Apparent Binding (%)					
	Human Plasma	Human Cervicovaginal Fluid	Ectocervix	Myometrium	Endometrium	Fallopian Tubes
DPV	99.06 ± 1.27	97.73 ± 1.31	98.27 ± 0.80	98.88 ± 0.16	97.49 ± 1.33	99.45 ± 0.02
LNG	97.67 ± 0.11	89.63 ± 1.31	92.42 ± 1.92	96.29 ± 0.76	97.08 ± 0.58	94.45 ± 1.47
MK-2048	96.12 ± 0.33	84.75 ± 3.00 *	98.61 ± 0.36	97.96 ± 0.54	98.46 ± 0.77	99.05 ± 0.25
EFdA	15.00 ± 10.88	27.52 ± 4.42	61.02 ± 13.04	62.10 ± 24.86	54.5 ± 19.45	52.14 ± 18.42

**Table 3:** Tissue permeability data of each model drug across human ectocervix, endometrium, myometrium, and fallopian tubes. Data are shown as the mean ± standard deviation from tissues obtained from 3–4 donors, with multiple technical replicates (*n* = 4–20) conducted per tissue type across donors. Statistical differences determined by Welch’s unpaired *t*-test on log10(P_app_). * = Data were obtained from Ref. [30]. § = Data were obtained from Ref. [15].

Tissue	Model Drug	DPV	LNG	MK-2048	EFdA
Ectocervix	N (replicates)	11	15	10	20
	P_app_ A to B (×10^−6^, cm/s)	0.48 ± 0.38	17.7 ± 7.84 ^§^	0.047 ± 0.029 *	8.73 ± 6.22
	N (replicates)	11	15	8	12
	P_app_ B to A (×10^−6^, cm/s)	0.45 ± 0.76	87.2 ± 60.4 ^§^	0.12 ± 0.074 *	7.29 ± 4.80
	Efflux Ratio	0.95	4.93	2.55	0.84
	*p*-value	0.213	<0.0001	0.009	0.746
Endometrium	N (replicates)	6	17	8	9
	P_app_ A to B (×10^−6^, cm/s)	0.087 ± 0.075	13 ± 8.15 ^§^	0.97 ± 0.85	6.92 ± 4.24
	N (replicates)	7	16	7	9
	P_app_ B to A (×10^−6^, cm/s)	0.068 ± 0.045	25.4 ± 10.7 ^§^	3.35 ± 2.19	4.28 ± 3.33
	Efflux Ratio	0.77	1.95	3.44	0.76
	*p*-value	0.938	0.0002	0.003	0.067
Myometrium	N (replicates)	12	18	11	8
	P_app_ A to B (×10^−6^, cm/s)	0.23 ± 0.17	13.1 ± 7.07 ^§^	0.99 ± 0.7	5.23 ± 2.66
Fallopian Tubes	N (replicates)	12	7	10	12
	P_app_ A to B (×10^−6^, cm/s)	0.08 ± 0.09	3.32 ± 3.10	1.09 ± 1.18	7.75 ± 7.26
	N (replicates)	8	6	12	12
	P_app_ B to A (×10^−6^, cm/s)	0.13 ± 0.14	2.62 ± 1.24	3.86 ± 3.14	4.03 ± 3.07
	Efflux Ratio	1.60	0.79	3.53	0.52
	*p*-value	0.412	0.835	0.0067	0.217

**Table 4:** Cell permeability of model drugs across Caco-2 monolayer and 3D vaginal model. Data are shown as the mean ± standard deviation. Statistical differences determined by two-way ANOVA on log10(P_app_).

In vitro model	Model Drug	DPV	LNG	MK-2048	EFdA
Caco-2 monolayer	N (replicates)	9	12	15	12
	P_app_ A to B (×10^−6^, cm/s)	3.68 ± 1.47	52.7 ± 16.8	39.3 ± 16.7	0.09 ± 0.26
	P_app_ B to A (×10^−6^, cm/s)	4.48 ± 2.93	32 ± 10.3	102 ± 75.8	6.13 ± 1.84
	Efflux Ratio	1.09 ± 0.48	0.64 ± 0.25	2.59 ± 1.77	7.14 ± 2.31
	*p*-value	0.965	0.013	<0.0001	<0.0001
3D vaginal model	N (replicates)	10	11	11	12
	P_app_ A to B (×10^−6^, cm/s)	6.17 ± 2.74	5.97 ± 1.09	63.7 ± 24.2	13.7 ± 2.09
	P_app_ B to A (×10^−6^, cm/s)	6.97 ± 2.81	4.84 ± 2.53	84.3 ± 37.8	12.3 ± 2.89
	Efflux Ratio	1.24 ± 0.87	0.81 ± 0.43	1.38 ± 0.54	0.89 ± 0.12
	*p*-value	0.527	0.333	0.422	0.897

## Data Availability

The authors declare that all the data supporting the findings of this study are available within the paper and its Supplemental Data.

## Supplementary Materials

The following supporting information can be downloaded at: https://www.mdpi.com/article/10.3390/pharmaceutics18070903/s1 [15,18,21,22,47,48,49], Table S1: Summary of LC-MS conditions for the model drugs; Table S2: Summary of LC-MS/MS performance metrics for all four model drugs; Table S3: Summary of final concentrations of each model drug used in permeability studies; Table S4: Solubility of each model drug in human pooled plasma. Data are shown as the mean ± standard deviation.
