# Supplementary material for: Determining Critical Physiological and Drug Specific Parameters for Enhancing a Physiologically Based Pharmacokinetics Model for the Female Reproductive Tract
Source: Pharmaceutics. 2026 Jul 22;18(7):903. doi: 10.3390/pharmaceutics18070903 (PMC13415130; doi:10.3390/pharmaceutics18070903)
Supplement: Supplementary file 1 [file pharmaceutics-18-00903-s001.zip › pharmaceutics-4362643-supplementary.pdf]

Determining critical physiological and drug specific parameters for enhancing a physiologically based pharmacokinetics model for the female reproductive tract

An Le[1,2], Riyazuddin Mohammed[2,4], Junmei Zhang[2,4], Lin Wang[1,2], Guru R. Valicherla[1,2], Phillip W. Graebing[1,2], Robert Bies[3], and Lisa C. Rohan[1,2,4*]

LC-MS/MS methods

The LC-MS/MS methods were adopted from the previously published work [15,18,21]. Detailed conditions that were specific to the individual model drugs are listed in Table S1. All injection volumes were 10µL. The calibration data was analyzed using the linear regression analysis with weighing factor (1/X) for the best fit line of y = mx + c. Linearity was established with eight concentrations. The criteria for acceptance of each back calculated calibration standards should be within 100 ± 15 %, except for the lower limit of quantification (LLOQ) which is within 100 ± 20 % deviation from the nominal value according to the USFDA guideline [22].

Table S1. Summary of LC-MS conditions for the model drugs

Model drug (internal standard)	Column Type	Mobile Phase	Flow rate/ Run Time	MRM transitions of model drug (internal standard)
DPV (d^4- DPV)	Waters Acquity UHPLC BEH C18 analytical column (1.7 µm, 2.1×100 mm)	A: 0.1% formic acid in water (v/v)<br><br>B: 100% acetonitrile<br><br>Ratio A:B of 60:40.	0.4mL/min 5 minutes	m/z 330.09 →158.06 (m/z 334.09 → 158.91)
MK-2048 (d^6- MK-2048)	Waters Acquity UHPLC BEH C18 analytical column (1.7 µm, 2.1×50 mm)		0.4mL/min 4 minutes	m/z 462 →143 (m/z 468 → 143)
LNG (d^6- LNG)	Waters Acquity UHPLC BEH C18 analytical column (1.7 µm, 2.1×150 mm)		0.4mL/min 4 minutes	m/z 313.10 → 245.16 (m/z 319.15 → 251.19)
EFdA ($^{13}C^{15}N_3$-EFdA)	Waters Acquity PREMIER HSS T3 analytical column (1.8 µm, 2.1×100 mm) with ACQUITY UPLC	A: 10% methanol in water and 0.1% formic acid	0.3mL/min 4 minutes	m/z 294.14 → 154.02 (m/z 298.10 →158)

	BEH C18 1.7μm guard column	B: 90% methanol in water and 0.1% formic acid<br><br>Ratio A:B 70:30		

The LC-MS/MS performance metrics are summarized in Table S2 for all four model drugs across all matrices. As shown in Table S2, all the calibration curves were linear ($R^2 \geq 0.99$) over the concentration range specified for each model drug. The LLOQ ranged from 0.05 to 1 ng/ml, which was sufficient for the intended purpose. Intra- and inter-day accuracy and precision were within ±12%, and ±10%, respectively, across three days.

Table S2. Summary of LC-MS/MS performance metrics for all four model drugs

Parameter	DPV (all matrices)	MK-2048 (all matrices)	LNG (all matrices)	EFdA (all matrices)
Calibrator range (ng/mL)	0.05 – 1000	0.3 – 3000	0.3 – 2000	1 – 1000
LLOQ (ng/mL)	0.05	0.3	0.3	1
Linearity (R^2)	0.991377 - 0.998911	0.989977 - 0.999496	0.993974 - 0.99950	0.990943 - 0.999692
Accuracy (%)	92.14 – 101.07	96.56 – 106.86	96.12 – 111.94	94.22 – 106.35
Precision (%)	1.61 – 7.16	1.71 – 7.31	1.42 – 8.65	5.01 – 9.79

Note: Linearity, accuracy, and precision were evaluated over three independent days across the calibration range for each matrix

In addition, the use of stable isotope-labeled internal standards was used to account for variability in extraction efficiency and potential matrix effects. Extraction efficiency was consistent (81–105%). This performance is likely achieved due to the use of stable isotope-labeled internal standards for all calibration standards, quality controls, and biological samples and the use of the actual blank matrix (plasma, cervicovaginal fluid, tissue homogenates, HBSS/KRB) for all calibration standards (except that PBS was used for cervicovaginal fluid; suitability was confirmed by comparing the peak area ratios and back calculated concentrations of quality controls prepared in PBS and cervicovaginal fluid, respectively) and quality controls. The experimental handling conditions were designed to minimize potential analyte degradation, supporting reliability of the LC-MS/MS methods utilized in this work.

Liquid-liquid extraction procedure

Briefly, 100 µl of samples from the donor wells were added to 100 µl 1X PBS and mixed well. 100 µl of samples from the receptor wells were added to 100 µl blank matrix and mixed well. 4 mL of methyl tert-butyl ethyl (MTBE) was added to each sample and vortexed at 1000 rpm for 10 minutes to precipitate the proteins. The samples were centrifuged at 10,000x g for 15 minutes. The aqueous layer was frozen by dipping the conical tubes in a tray with 70% ethanol containing dry ice. 3.6 mL of MTBE was aliquoted in a glass tube and evaporated in a vacuum evaporator for 1 hour at 60 ^{o}C. The pellet was reconstituted with 200 µl of mobile phase and vortexed for 5 minutes at 1000 rpm. The samples were then analyzed by LC-MS/MS as described above. Peak area ratios (analyte peak area/IS peak area) were utilized to calculate apparent binding.

Cell culture set up for cell permeability studies

Caco-2 cells were seeded at a density of 75,000 cells/cm^2 onto each polycarbonate insert (1.12 cm^2, 0.4 µm, Corning) in 12-well tissue culture plates and were allowed to grow and differentiate for 21 days. The cell growth medium was changed every other day.

A 3D vaginal epithelial (VK2/BJ) cell model was prepared by initial collagen coating, then seeding BJ fibroblast cells on the basal side and finally seeding VK2/E6E7 cells on the apical side and incubating for the formation of a 3D vaginal model. Initial collagen coating was achieved by inverting the transwell inserts (0.332 cm^2, 3 µm, Corning) and adding rat tail collagen I (10 µg/cm^2) diluted in 0.02 N acetic acid on the basal side. Later, BJ fibroblast cells were seeded (30,000 cells) on the collagen coated basal side of the transwell inserts and allowed to attach for 6 hrs. After 48 hrs of seeding fibroblast cells, VK2/E6E7 cells were seeded (0.1 million cells) on the apical side of the transwell inserts and allowed to differentiate for 8 days. During this period, an epithelial–air interface was created by adding fresh media every alternate day only on the basal side.

Table S3. Summary of final concentrations of each model drug used in permeability studies.

Model drug	Final concentration used in tissue permeability studies	Final concentration used in cell permeability studies
DPV	303.6 μM	5 μM
MK-2048	100 μM	1.5 μM
LNG	62.2 μM	16 μM
EFdA	255.7 μM	42.6 μM

Table S4. Solubility of each model drug in human pooled plasma. Data are shown as the mean±standard deviation.

Model drug	Human plasma solubility (μg/mL, 24 hours, n=3 replicates)	Aqueous solubility at physiological pH (μg/mL)*
Dapivirine	34.14 ± 1.04	<1 [47]
LNG	66.38 ± 10.46	~2 [48]
MK-2048	456.92 ± 38.59	~20.392 ± 2.734 [18]
EFdA	1808.02 ± 67.36	~800-900 [49]

*The aqueous solubility data listed in this data was obtained from available literature. The experimental conditions used to obtain aqueous solubility differed from one model drug to another. However, it is apparent all four model drugs have higher solubility in plasma than in aqueous buffers; this solubility enhancement is more significant for hydrophobic drugs.

References:

15. Li, X., et al., *Mechanism-Based Predictions of Local Tissue and Systemic Exposure for Drug Products Delivered Through the Female Reproductive Tract.* J Clin Pharmacol, 2026. **66**(2).
18. Zheng, R., et al., *In Vitro Pharmacokinetic Properties of MK-2048, a Potent Drug Candidate for HIV Prevention.* Viruses, 2026. **18**(5): p. 561.
21. Valicherla, G.R.; Graebing, P.; Zhang,, J.; Zheng, R.; Nuttall, J.; Silvera , P.; Rohan, L.C. Investigating the Contribution of Drug-Metabolizing Enzymes in Drug-Drug Interactions of Dapivirine and Miconazole. *Pharmaceutics* **2021**, 13, 2193.
22. USFDA, *Bioanalytical Method Validation Guidance for Industry.* 2018: Silver Spring, MD, USA.

47. Fetherston, S. M., et al. *Partial protection against multiple RT-SHIV162P3 vaginal challenge of rhesus macaques by a silicone elastomer vaginal ring releasing the NNRTI MC1220.* Journal of Antimicrobial Chemotherapy, 2013. *68*(2), 394–403. https://doi.org/10.1093/jac/dks415
48. Levonorgestrel. https://pubchem.ncbi.nlm.nih.gov/compound/Levonorgestrel. Last Accessed July 8, 2026.
49. Zhang, W., Parniak, M. A., Mitsuya, H., Sarafianos, S. G., Graebing, P. W., & Rohan, L. C. *Preformulation studies of EFdA, a novel nucleoside reverse transcriptase inhibitor for HIV prevention.* Drug Development and Industrial Pharmacy, 2014. *40*(8), 1101–1111. https://doi.org/10.3109/03639045.2013.809535
